# Tools for Etiologic Diagnosis of Drug-Induced Allergic Conditions

**DOI:** 10.3390/ijms241612577

**Published:** 2023-08-08

**Authors:** Rosa Rodríguez-Pérez, Leticia de las Vecillas, Rosario Cabañas, Teresa Bellón

**Affiliations:** 1 Institute for Health Research Hospital Universitario La Paz (IdiPAZ), Paseo Castellana 261, 28046 Madrid, Spain; leticia.vecillas@salud.madrid.org (L.d.l.V.); mrosario.cabanas@salud.madrid.org (R.C.); teresa.bellon@salud.madrid.org (T.B.); 2 Allergy Department, La Paz University Hospital, Paseo Castellana 261, 28046 Madrid, Spain; 3 PIELenRed Consortium, 28046 Madrid, Spain; 4 Center for Biomedical Research Network on Rare Diseases (CIBERER U754), 28046 Madrid, Spain

**Keywords:** drug hypersensitivity, in vitro tests, diagnostic, IgE, T cells, anaphylaxis, severe cutaneous reactions

## Abstract

Drug hypersensitivity reactions are a serious concern in clinical practice because they can be severe and result in lifelong sequelae. An accurate diagnosis and identification of the culprit drug is essential to prevent future reactions as well as for the identification of safe treatment alternatives. Nonetheless, the diagnosis can be challenging. In vivo and in vitro tests can be helpful, although none are conclusive; therefore, the tests are not usually performed in isolation but as part of a diagnostic algorithm. In addition, some in vitro tests are only available in research laboratories, and standardization has not been fully accomplished. Collaborating research is needed to improve drug hypersensitivity reaction diagnosis. In this review, we update the current available in vivo and in vitro tools with their pros and cons and propose an algorithm to integrate them into clinical practice.

## 1. Introduction

Drug hypersensitivity reactions (DHRs) are adverse effects of pharmaceutical formulations (including active drugs and excipients) that clinically resemble allergies. DHRs can be allergic or non-allergic in nature, with drug allergies being immunologically mediated DHRs. The term non-allergic hypersensitivity is preferred for those reactions in which the underlying pathogenic mechanisms do not involve a B cell-mediated or T cell-mediated drug-specific immune response, which have also been referred to as anaphylactoid or pseudoallergic by several authors [1,2]. However, B and T cell-specific immune mechanisms are not always easily identified, even in allergic reactions.

In this review, we will focus on drug allergic reactions, specially on the tools available for the allergological workup used to gather evidence supporting specific immune responses and for identification of inciting drugs in immunoglobulin IgE- and T cell-mediated allergies.

## 2. Drug Hypersensitivity Reactions: Clinical Classification and Phenotypes

The classification of drug allergic reactions is challenging. From the clinical point of view, DHRs are usually classified as immediate or non-immediate (delayed) depending on the time of onset during treatment [2]. Immediate reactions typically appear within 1–6 h after the last drug administration; non-immediate reactions take place any time from 1 h after the initial drug administration [2]. They commonly occur after several days of treatment and are often associated with a delayed T cell-dependent type of allergic mechanism [2], although IgG/IgM and immune complex-mediated diseases can also manifest as delayed hypersensitivity. Non-immediate reactions can also occur more rapidly after re-exposure in patients with prior adverse events.

### 2.1. Immediate Drug Hypersensitivity Reactions

Immediate allergic DHRs are induced by an IgE-mediated mechanism that activates mast cells and basophils in an antigen-specific manner through the high-affinity IgE receptor (FcεRI) expressed on their cell membrane. This activation promotes the release of preformed mediators (mainly tryptase and histamine), inducing symptoms within minutes, and the production of new mediators (such as leukotrienes, prostaglandins, and cytokines) [2].

Symptoms induced by the activation of mast cells and basophils range from mild exanthemas and urticaria/hives, flushing, pruritus, angioedema, gastrointestinal, and respiratory symptoms (rhinorrhea, dyspnea, bronchospasm) to cardiovascular involvement (tachycardia, hypotension) and life-threatening reactions such as severe anaphylaxis [2]. Recognition of anaphylaxis can be difficult, given that different reaction patterns could cause clinical uncertainty. A detailed assessment of clinical reaction features and severity grading might help in diagnosis [3].

Recently, immediate reactions induced by specific IgG anti-drug antibodies (ADAs) have been described [4], called ADA-mediated reactions [5]. The cytokine release induced by some specific drugs, such as chemotherapeutics and biologics, is also related to the development of immediate DHRs, termed cytokine release reactions, with symptoms differing from those triggered by mast cell/basophil activation, including fever, chills, pain (head, back, chest), rigors, and desaturation [5]. When symptoms of both ADA-mediated reactions and cytokine release reactions concur, the reactions are called mixed reactions [5].

### 2.2. Non-Immediate/Delayed Allergic Drug Hypersensitivity Reactions

Non-immediate or delayed allergic DHRs can manifest with variable cutaneous symptoms, such as delayed urticaria, maculopapular eruptions, fixed drug eruptions (FDEs), vasculitis, Stevens-Johnson syndrome/toxic epidermal necrolysis (SJS/TEN), generalized bullous fixed drug eruptions, drug reaction with eosinophilia and systemic symptoms (DRESS), acute generalized exanthematous pustulosis (AGEP), and symmetrical drug-related intertriginous and flexural exanthemas (SDRIFE). Internal organs can be affected either alone or with cutaneous symptoms (hypersensitivity syndrome (HSS)/DRESS/drug-induced hypersensitivity, vasculitis, SJS/TEN) and can include hepatitis, renal failure, pneumonitis, anemia, neutropenia, and thrombocytopenia [2].

DRESS, SJS/TEN, and AGEP are considered severe cutaneous adverse reactions (SCARs) to drugs due to their high morbidity and mortality [6], with SJS/TEN being the most severe form of SCAR [7], with an overall mortality of 34% at one year post-reaction [8].

Accurate diagnosis of specific clinical entities is key for identification of potential culprits. An exhaustive description of criteria for differential diagnosis is beyond the scope of this review. Details on this topic can be found in several previous publications [7,9,10,11,12].

## 3. Pathogenesis and Pathophysiology

Adverse drug reactions (ADRs) are due to various mechanisms. Rawlings and Thompson proposed a first classification including two groups of ADRs: type A and type B [13]. Type A reactions are due to the pharmacological activity of the drug; they can occur in every individual and are predictable to some extent. Type B reactions are less well defined and comprise approximately 15% of all adverse reactions. Allergic DHRs are a subgroup of type B reactions. In recent classifications, Type A reactions are labeled as “on-target” reactions, whereas type B are referred to as “off-target” adverse reactions [14].

As previously mentioned, the most accepted clinical classification of DHRs takes into account the time between the start of treatment and the onset of symptoms.

**Figure 1:**
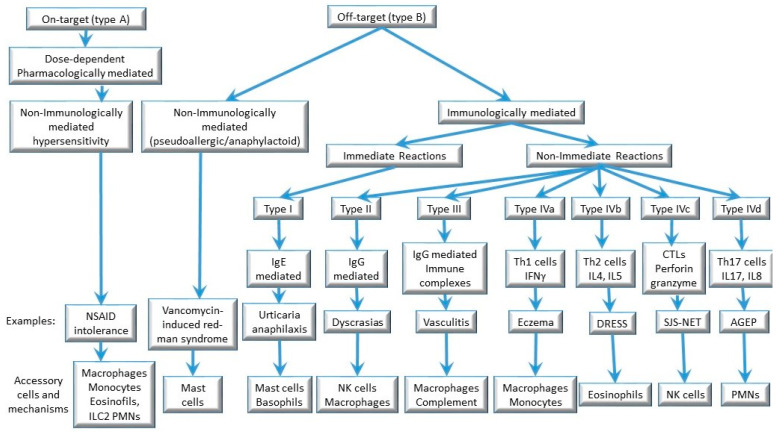
Mechanisms involved in drug hypersensitivity reactions.

The classification of hypersensitivity reactions proposed by Gell and Coombs links the clinical phenotype to the immune mechanism involved. The immediate symptoms (urticaria, anaphylaxis) are categorized as type I hypersensitivity and are caused by allergen-specific IgE and mast cell degranulation. Delayed symptoms (e.g., exanthemas, hepatitis, Stevens-Johnson Syndrome/toxic epidermal necrolysis, DRESS) that are dependent on the activation of drug-specific T cells are classified as type IV hypersensitivity reactions. Some delayed reactions, such as hemolysis, thrombocytopenia, arthralgia, and vasculitis, could be due to drug-induced IgG/IgM antibodies (type II and type III hypersensitivity) and are less frequent [15,16] (Figure 1). We will focus on Type I and Type IV hypersensitivity reactions because these are the most frequently observed in allergy clinics.

Most medications are small molecular weight compounds with simple chemical structures that are not easily recognized by the immune system so as to elicit a primary immune response involving specific T or B cells. Thus, most drugs are not effective immunogens. However, some drugs (or their metabolites) can become immunogenic after covalent binding to host proteins (e.g., albumin). The drug is then referred to as a “hapten”, the host protein as a “carrier”, and the complex or conjugate as “hapten–carrier”. Hapten–carrier conjugates are immunogenic for B cells (antibody responses) and for T cells. Drugs that cannot act directly as haptens but give rise to reactive metabolites are referred to as prohaptens.

Drugs with the capability to haptenate host proteins are penicillins and other beta-lactam antibiotics (cephalosporins, carbapenems, and monobactams), sulfonamides, metamizole, quinolones, radiocontrast media, and muscle relaxants (rocuronium, succinylcholine), although the list is not exhaustive. Drugs that can generate reactive metabolites (prohaptens) are sulfonamide antimicrobials (sulfamethoxazole), phenacetin, or halothane, phenytoin, carbamazepine, and lamotrigine (although aromatic anticonvulsants are not common elicitors of IgE response). Also, several drugs can behave as a hapten, and their metabolites also have this capacity (e.g., metamizole). Recombinant proteins such as monoclonal antibodies, solubilized receptors and cytokines, insulin and other hormones, enzymes, protamine, antisera, and vaccines are pharmaceutical products that can elicit an antibody response by themselves because they are peptides or proteins (true allergens or immunogens) [17]. Hapten–carrier complexes are processed by antigen-presenting cells, and the haptenated peptides are then presented to T cells via human leukocyte antigen (HLA) molecules, which are recognized by T cell receptors.

### 3.1. Immediate IgE-Mediated Allergic Reactions (Type I Reactions)

Various mechanisms and pathways can be involved in mast cell activation that lead to immediate hypersensitivity symptoms, including immunoglobulin-mediated and direct mast cell activation. Symptoms are similar, independent of the underlying mechanism involved and caused by the release of mediators such as histamine, tryptase, platelet-activating factor (PAF), and cysteinyl leukotrienes. Histamine induces smooth muscle constriction and increases vascular permeability, leading to flushing, pruritus, rhinorrhea, tachycardia, and bronchospasm. Tryptase activates the complement cascade, the coagulation pathway, and the kallikrein–kinin system, contributing to the development of hypotension and angioedema. PAF and cysteinyl leukotrienes also enhance vascular permeability, leading to hypotension [18]. Type I reactions require the presence of drug-specific immunoglobulin IgE.

The formation of drug-specific IgE starts with the sensitization stage, including the activation of Th2 and Tfh cells by the drug. B and T cell interactions allow the production of primary response of antibodies (IgM). Upon antigen encounter and B and T cell activation, CD40-CD40L interactions, in conjunction with interleukin (IL)-21 and Th2 cytokines, enable further immunoglobulin class-switching [19,20]. The B cells then proliferate, mature, and differentiate into IgE-secreting plasma cells. Drug-specific IgE molecules diffuse through the circulation and attach to the high-affinity IgE receptor (FcεRI) on the surface of mast cells and basophils throughout the body.

When an individual is re-exposed to the medication, the effector stage is induced. During the effector stage, the drug–carrier complex binds to drug-specific IgE on the surface of mast cells and/or basophils. Consequently, the cross-linking of two or more high-affinity IgE receptors results in a sudden and widespread activation and release of an array of vasoactive mediators (e.g., histamine, prostaglandins, leukotrienes) [21].

Sensitization and effector stages can also occur during the same treatment course if it lasts long enough.

Beta-lactam antibiotics are considered the primary hapten triggers of IgE-mediated anaphylaxis induced by drugs [22]. An additional mechanism by which IgE-mediated reactions arise is previous sensitization to a cross-reacting agent, e.g., sensitization to quaternary ammonium compounds in cosmetic and personal care products, leading to type I reactions to neuromuscular-blocking agents used in anesthesia induction, which contain tertiary and quaternary substituted ammonium structures [17].

### 3.2. Non-Immediate T Cell-Mediated Allergic Reactions (Type IV Reactions)

Type IV hypersensitivity reactions are induced by activated T cells, with participation of both CD4+ and CD8+ T cells. Advances in the understanding of T cell functions led to a sub-classification of T cell-mediated hypersensitivity reactions into three subgroups (types IVa, IVb, IVc) [23]. A fourth subgroup, type IVd, was later added to sub-categorize type IV reactions into types IVa–IVd according to the dominant cytokines and to the contribution of certain subpopulations of leukocytes to skin inflammation and tissue damage [24] (Figure 1).

In this classification, type IVa reactions involve the actions of CD4+ T helper (Th)1 cells. Type IVb reactions correspond to Th2 responses with production of IL-4, IL-13, and IL-5, which facilitate eosinophilic inflammation. In type IVc reactions, cytotoxic CD8+ T cells are the main effectors of tissue injury, with contribution of natural killer cells. Lastly, in type IVd reactions, T cells secreting IL-8/CXCL8 promote neutrophilia as well as neutrophil recruitment to affected tissue [24]. Traditionally, DRESS is considered a type IVb Th2-driven reaction, SJS/TEN is a type IVc cytotoxic reaction, and AGEP is a type IVd reaction [25,26] (Figure 1). Although this classification might be useful, there is overlap between the subtypes, which are not mutually exclusive. For example, high interferon (IFN)-γ levels have been identified in serum and blister fluid from patients with SJS/TEN [27], and drug-specific CD8+ cytotoxic T cells can also be involved in DRESS and AGEP [28]. Of note, any of these reactions can occur in response to any drug, meaning that similar immune mechanisms are triggered in response to different chemical compounds.

T cells recognize the antigen through their antigen-specific T cell receptors (TCRs). Somatic recombination of the TCR genes can lead to a unique, randomly generated repertoire of TCR specificities in each individual [29]. To be stimulated, TCRs must bind a bimolecular complex displayed at the surface of the antigen-presenting (or target) cells, consisting of a fragment of a protein antigen (peptide) bound in the peptide-binding cleft of an HLA molecule. The genes coding HLA molecules are clustered together in the major histocompatibility complex (MHC) in chromosome 6 in humans. Given that the MHC is the most polymorphic region in the human genome, the available HLA haplotypes tend to be specific for each individual. The repertoire of available TCRs, together with the available HLA haplotype in each individual, limits the possibilities for developing specific adaptive immune cell responses to encountered antigens. The occurrence of a DHR needs the conjunction of several risk factors, including the availability of adequate HLA and TCR variants (Jackpot theory) [30].

Various models have been proposed for xenobiotic drug recognition by specific TCRs. The first model considers the fact that some drugs behave as haptens, as explained above. Hapten–protein conjugates can elicit hapten-specific adaptive immune responses because the hapten can be recognized by specific TCRs after covalent binding to protein-derived peptides presented by HLA molecules. However, in vitro development of drug-specific T cell clones has led to the finding that some lymphocytes can be stimulated in the presence of specific drugs and HLA molecules in the absence of antigen-presenting cells’ active metabolism. This led to the hypothesis of non-covalent interaction between TCR, the HLA–peptide complex, and the drug (no need for haptenization), and the development of the so-called “pharmacological interaction (p-i)” model [31]. Any drug can stimulate specific T cells through this mechanism. More recently, it has been demonstrated that the anti-retroviral drug, abacavir, can accommodate itself within one of the pockets of the peptide-binding cleft, specifically in HLA-B*57:01, changing its conformation and allowing the binding of a new repertoire of peptides. Given that this new peptide repertoire is not found in the thymus at the moment of thymic selection, T cells autoreactive to these new peptides presented by HLA-B*57:01 “filled” with abacavir can be found in the periphery and can behave as alloreactive T cells, leading to the development of hypersensitivity reactions to abacavir in carriers of the risk allele HLA-B*57:01 [32,33,34]. This is known as the altered peptide model (Reviewed in [35]).

Although primary immune responses are well demonstrated for xenobiotics behaving as haptens, the tendency for some drug reactions to occur with short latency periods while maintaining long-lasting memory T cell responses suggests the absence of a sensitization or priming phase. It also suggests that cross-reactive memory T cells could be important in individuals exposed to a drug for the first time. As an example, abacavir-reacting memory CD8+ T cells have been identified in unexposed individuals, suggesting priming by earlier exposure to another antigen [36]. This phenomenon is known as heterologous immunity [37], and it is not mutually exclusive to other models of T cell activation in drug hypersensitivity reactions [38].

## 4. Etiologic Diagnosis: Identification of the Culprit Drug

### 4.1. Clinical History

The first step in the diagnosis approach to drug allergy is to suspect a hypersensitivity reaction to an administered drug. A precise description of the morphology and chronology of the reaction is mandatory [2]. Thorough anamnesis must include the patient’s history of drug exposure (dosage, time of intake, interval between beginning and ending intake, and start of skin or any other clinical manifestations) [39].

In the case of immediate itching reactions, appearance of hives/angioedema, rash, dyspnea, hypotension, and other systemic symptoms must be recorded [40].

As for non-immediate reactions, we must document the type of skin symptoms (if they have occurred), such as blisters, skin pain, Nikolsky’s sign, and morphology of the exanthema or erythema, together with the existence of eosinophilia, additional hematological alterations, fever, liver injury, or other organic symptoms.

Treatment of patients who develop signs/symptoms of drug allergy while receiving multiple medications simultaneously is best approached in a systematic manner. Data should ideally be recorded in a uniform format, and in order to harmonize the DHR diagnostic procedures, members of European Academy of Allergy and Clinical Immunology (EAACI)-Drug Allergy Interest Group/European Network on Drug Allergy have developed a questionnaire available in several languages that can be useful as a guide [39].

A timeline chart should be constructed to bring together all available information, such as a precise timeline of medication intake and the appearance and resolution of symptoms, including specific features of resolution such as desquamation, residual hyperpigmentation, or any sequelae [40,41]. Photographs of the cutaneous lesions, if not present at the time of the interview, can provide important information. During the acute reaction, clinical pictures, covering the entire body whenever possible, should be taken because they can be helpful for further diagnosis.

The patient’s medical background, previous drug allergy reactions, and family history of DHRs with a specific drug can be relevant [42]. Essential also in the clinical history is the tolerance to other drugs of the same class taken since the reaction. Concurrent medications should always be recorded. Nonsteroidal anti-inflammatory drugs (NSAIDs) and other conditions that can work as cofactors (such as alcohol intake, menstruation, and exercise) in immediate reactions are important to register [43].

The medical records should be reviewed, if possible. In critically ill patients who cannot communicate, these could be the only available source of information other than that provided by the patient’s family.

The diagnosis of drug allergic reactions also requires knowledge of the scientific literature, including access to Medline searches to look up a particular compound and a specific hypersensitivity reaction. The literature can be especially relevant in the case of SCARs and in reactions with new drugs that have recently been marketed.

History alone is often insufficient for establishing current drug sensitization, given that it could be limited by the patient’s recall and interpretation of his/her symptoms, which can be subjective [44].

After the carefully taken anamnesis, the allergists will know whether the reaction they are dealing with is a probable immediate or non-immediate drug allergic reaction, its phenotype, and which tools should be used to identify the culprit drug.

During the acute phase of the reaction, there is general agreement to not perform skin testing [26]. The only tools used to identify the culprit drug will be the clinical history and the algorithms of causality assessment, which are of great relevance in SCARs [34] and other DHRs.

### 4.2. Pharmacovigilance Algorithms

Various methods have been proposed to evaluate the causal relationship between an adverse event and the medication taken by the patient [45]. The algorithm methods are primarily based on decision trees or consecutive answers to specific questions, resulting in a sum of scores [46]. Algorithms give structured and standardized methods of assessment in a systematic approach to evaluating causal associations in adverse drug reactions based on parameters such as time to onset of the adverse event or temporal sequence, previous adverse drug reaction history, and response to drug withdrawal or rechallenge.

Causality algorithms present high (near 100%) sensitivity and positive predictive value (PPV) but low (below 50%) specificity and negative predictive value (NPV) [47]. The Naranjo algorithm is mostly used in English-speaking countries [48]; Bégaud’s algorithm is applied in the French-speaking world [49], and Spanish-speaking countries use the algorithm of the Spanish pharmacovigilance system [50]. All are modifications of the algorithm developed by Karch and Lasagna [51], with few variations in the sets of questions associated with scores for calculating the likelihood of a cause–effect relationship. Most algorithms evaluate chronology, the degree of knowledge of the relationship between the drug and the specific reaction, the patient’s response to the drug’s withdrawal, the rechallenge effect, and possible alternative causes. The final evaluation is listed as improbable, conditional, possible, probable, or defined. The final case evaluation of each drug is usually listed as not related (if unrelated or conditional) or related (if possible, probable, or defined) [52].

The main caveat when using these algorithms is that they were primarily developed to assess drug causality in type A (on-target) adverse drug reactions, with drug allergies being mostly type B (off-target) reactions and some severe, in which re-exposures are largely contraindicated. It is often not possible to answer some of the questions proposed for scoring, such as finding toxic levels of the drug in plasma, the effect of increasing doses, or the response to a drug rechallenge, all of which are included in the Naranjo algorithm. In addition, their usefulness during the acute phase, when it is urgent to identify the culprit drug, is quite limited, although they can be of particular help in polymedicated patients.

The RegiSCAR group used their own registry to build a specific algorithm denominated ALDEN (algorithm of drug causality in epidermal necrolysis) for drug causality assessment in patients with SJS/TEN based on the results of two case–control studies. It is a six-item scoring system validated for community-acquired cases [53]. ALDEN is more sensitive than a general pharmacovigilance algorithm and is recognized as one of the most reliable tools for identifying culprit drugs for SJS/TEN [54]. It assigns a probability category to each drug: very unlikely, unlikely, possible, probable, or very probable. Only drugs classified as probable or very probable are considered as related [55]. ALDEN is generally used as a tool for retrospective assessment of drug causality in SJS/TEN [56]; however, it can also be helpful in the acute phase of illness when the utility of the allergy workup is rather low.

### 4.3. Value and Limitations of In Vivo Tests

Although drug challenge is the gold standard technique to confirm or discard a drug allergy, due to safety concerns, it is used when all the available alternative tests (in vivo and in vitro) are negative, considering the patient’s comorbidities and the necessity and utility of achieving an accurate diagnosis.

Skin tests (prick and intradermal) and patch testing are useful in vivo tools used for etiologic diagnosis of drug allergy. They are limited by low NPVs for some drugs; however, they can help to uncover possible cross-reactivity and find safe alternatives to the culprit drug. Their value is linked to the appropriate performance of the technique as well as the time interval between the reaction and the test, the type of hypersensitivity reaction, and the drugs being evaluated. Nevertheless, they should be conducted by professionals who are familiar with these techniques.

#### 4.3.1. Skin Tests: Prick and Intradermal Tests

Skin testing is the primary diagnostic tool for identifying patients with IgE- or T cell-mediated reactions to drugs [57,58]. It has the advantages of being relatively non-invasive, inexpensive to perform, provides rapid results, and is available in every allergy center [44].

There is wide variability in its performance and interpretation among the different centers and countries. To ensure the results are reproducible and accurate, skin testing must be performed in a consistent standardized fashion [44]. An international effort is being made to reduce this variability [26,59,60].

Skin tests can be particularly helpful for guiding drug challenges [42,57,61] and for characterizing the phenotype in reactions induced by biologicals [5] and chemotherapy drugs [62].

In terms of non-immediate reactions, with regard to the NPV of skin tests, a meta-analysis and large studies have underlined the usefulness of skin tests for identifying safe alternatives [26,63,64].

Skin testing has limitations and concerns. Its value depends on correctly performing the technique, using proper non-irritant drug concentrations, adequate interpretation of the results, adequate time to perform the tests and on the drug itself, and the clinical entity of the drug allergic reaction.

- **Technique and interpretation of the results**

**Prick test:** The test is performed on the volar surface of the forearm [57,65,66]. Adequate negative and positive controls must be used [57].

Some treatments can alter the results. Therefore, the drugs and treatments the patient is receiving should be reviewed before performing skin tests and stopped if needed, according to current guidelines [58,65].

This technique can be performed with almost all drugs, including those in which only solid presentation is available, except some opiates and some chemotherapeutic compounds [57,62].

The reading of prick tests is performed at 20 min; the results are considered positive if the papule (wheal) is ≥the negative control plus 3 mm and if there is a surrounding erythema [57,66].

Prick tests are mainly useful in the evaluation of immediate hypersensitivity and can occasionally be of value in the study of non-immediate reactions [57,67]. They are safer than intradermal tests (IDTs), albeit less sensitive. Therefore, they should be performed prior to IDTs for safety reasons.

**Intradermal test:** A small amount of drug is injected into the dermis when performing an IDT. Although an immediate wheal and flare reaction appears when an immediate drug allergy reaction occurs, it can also result in a delayed positive reading (infiltrated erythematous reaction) in case of non-immediate reactions [57,58,66]. It can appear in the following hours and even within 1 week after an IDT.

For IDTs, sterile injectable solutions are mandatory. Performing a positive control with histamine is not mandatory if a positive control prick test is performed. As negative controls, normal saline and/or any other solvent used to dilute the investigated drugs must be used [58].

EAACI guidelines for performing IDTs have recently been published [60].

- **Non-irritant drug concentrations**

Medications that directly activate mast cells cannot be studied by skin testing. In addition, the solvent used for drug dilution during testing should not be a skin irritant.

Non-irritating drug concentrations have been recommended for prick and intradermal testing in various position papers [58,59], including perioperative agents [68], radiocontrast media [69], chemotherapy [62], and biologicals [5]. In case of new drugs, it is recommended to test 10 control subjects in order to ensure specific results [58].

- **Time to perform skin tests**

It is generally agreed to perform skin tests at least 4 weeks after resolution and within 1 year after the drug allergy reaction [26,58,61,65,66,70,71].

Of note, IgE-mediated hypersensitivity to beta-lactams and other drugs, such as platinum compounds and iodinated contras media, can wane over time [70,71,72,73]. On the other hand, T cell-mediated hypersensitivity appears to be a long-lasting condition [74].

In DRESS, skin tests have been recommended to be performed at least 6 months after the disappearance of the cutaneous ADR [26,61,67]. However, other authors have not found any adverse reaction at earlier time points [75].

- **Value of skin tests according to the drug and clinical entity**

The value of skin testing depends on the specific drug. Skin tests are very useful in diagnosing immediate hypersensitivity reactions induced by beta-lactams [61], iodinated radiocontrast media [69], proton pump inhibitors [76], high molecular weight heparins [77], corticosteroids [68,78,79,80,81], and platinum compounds [62]. Although less frequently reported, they can also be useful in the study of reactions to other antibiotics, such as clindamycin [82,83] or isoniazid [84,85]. IDT with quinolones can yield false positive results [86,87]. Skin tests with excipients (mainly carboxymethylcellulose, polysorbate, and polyethylene glycol) can be useful [78,88,89,90]; however, special precaution is recommended with IDTs in patients with anaphylaxis [91]. Skin tests with gadolinium-based contrast agents [92], insulin [93], and chlorhexidine [94] can also be helpful, although chlorhexidine can yield false positive results.

Skin testing with vaccines is not standardized. False positive results are often found in delayed readings and should not be considered [58]. A prick-to-prick test with COVID-19 vaccines has proven useful to study immediate reactions [78,90,95].

Positive skin testing has also been reported for patients with hypersensitivity reactions to biologicals such as rituximab, anti-TNF agents, trastuzumab, and tocilizumab [96,97,98,99,100,101,102]. As for the NPV of skin tests, most available data are related to iodinated contrast media and beta-lactam antibiotics. The NPV is estimated at 98% for penicillin allergy [103] and approximately 90% for beta-lactams [61]. For iodinated contrast media, a range from 80% to 97.3% has been reported in different studies [63,69].

SCARs deserve special considerations. The value and safety of skin tests is controversial depending on the clinical entity analyzed [26]. IDTs with delayed reading have been shown to be useful for determining the responsible drug in patients with AGEP and negative patch tests [67,104,105]. They were also helpful in the diagnosis of TEN cases caused by beta-lactam antibiotics [106,107] and in a large series of patients with SJS/TEN [108]. Data on the utility of prick and/or IDTs in DRESS have also been reported [67,84,109,110,111], including in children [112]. Guidelines have been published for the management and diagnosis of DRESS syndrome, including prick and intradermal tests [41]. A systematic review supports the safety of skin tests as part of the diagnostic workup in DRESS [113].

- **Safety of skin testing in immediate and non-immediate reactions**

Skin testing must be adapted to the patient’s risk profile. The severity of the index reaction, comorbidities, and actual treatment of the patient must be considered. In immediate life-threatening reactions or in high-risk patients, specific IgE determination and basophil activation tests, if available, should be performed before starting the workup with in vivo tests [114].

The procedure employed to perform skin tests in the diagnosis of beta-lactam drug allergy has been well established [61]. The same protocols can be adapted when studying anaphylactic reactions due to other drugs. It is advisable to perform intradermal tests in a hospital setting [58].

In low-risk patients, SPTs and IDTs can be performed directly with the highest non-irritating concentrations [61].

In patients with non-immediate reactions, mild flare-ups with IDTs appear to be rare but possible [69]. In case of severe non-immediate reactions, performing in vitro tests (LTT, ELISpot) before in vivo tests has been recommended [41,42,61,114]. If the in vitro study has a negative result or if in vitro tests are not available, epicutaneous tests should precede skin testing [41,42,108] because, although better tolerated than presumed [111], there is a theoretical risk of eliciting a relapse of the initial cutaneous ADR, especially when performing intradermal tests [66]. For IDTs in SCARs, a stepwise protocol has been proposed [65,115]. Specific guidelines have been published for DRESS [41] and SJS/TEN [108]. However, the evidence supporting safety of intradermal tests in SJS/TEN is scarce. Until more evidence is available, they should only be performed in exceptional circumstances with the culprit drug while considering the risk and benefit [42,108].

A local reaction when performing IDT with glycopeptide drugs [67] has been reported. Taking special precautions when performing skin tests in patients with HIV has been recommended [42] as systemic reactions have been published [110], although other authors did not find any reaction in similar studies [75].

Nonetheless, in some publications, intradermal tests appear contraindicated in SCARs [60].

#### 4.3.2. Epicutaneous Tests

- **Technique and interpretation**

The epicutaneous test (or patch test) is a valuable diagnostic tool to identify the culprit drug after delayed DHRs. There is variation in its sensitivity and specificity depending on the reaction and the drug being investigated. It should be performed from 3–6 weeks to 3–6 months after the resolution of the reaction to avoid reactivation of the symptoms and false negative results, respectively [116,117]. Some treatments interfere with the patch test result and should be avoided before its performance (Table 1) [57].

Patch tests are preferably performed on the upper back. Sometimes, if the area is affected or the patient has suffered a severe cutaneous reaction, the outer surface of the forearm can be used (Table 1) [118]. Different sensitivity can depend on the anatomic patch site, which must be considered when evaluating the results [119].

The technique is based on the application of the allergen to the skin in an occlusive manner for 48 h, after which the patch test chambers are removed. Then, a first reading is performed (day 2), with a final reading between the fourth and seventh day (Table 1) [58,118].

The results are expressed the same as in allergic contact dermatitis (Table 1) [57].

Some drugs are commercially available to be tested in a dilution of 10% petrolatum. However, most must be prepared at the clinic, directly placed in the chamber when liquid, or mixed in petrolatum at 10% to 30% concentration. [57,118]. Other vehicles used for specific drugs are ethyl alcohol or dimethylsulfoxide [57,120,121].

When allergic reactions have been reported due to exposure to ultraviolet radiation, a photo patch might be useful (Table 1). The result is found by comparing the positive or negative reaction in both areas [57].

**Table 1:** Epicutaneous test technical recommendations.

Time	3–6 weeks to 3–6 months after the resolution of the reaction [116,117]
Vehicles [57,118,120,121]	Liquid drugs: placed directly in the chamber Petrolatum (10–30% concentration): Lesser concentrations to prevent false positive result: celecoxib, some formulations of colchicine, valproate, misoprostol, diltiazem, or chloroquine pills Ethyl alcohol: corticosteroids, cotrimoxazole Dimethylsulfoxide: benznidazole
Reading	Application: day 1 (D1) 1st reading: day 2 (D2)—(30 min after removing the test material) Last reading: day 5 to 7 (corticosteroids and aminoglycosides) [118]
Localization	Upper back (preferred) Outer surface of the forearm Postlesional skin (FDE)
Drugs to be suspended before the test	Topical corticosteroids: 7 days before Systemic corticosteroids, ultraviolet (UV) exposure and immunosuppressive therapies: one month before Antihistamines: do not interfere
Results	Erythema and papules (+) Erythema, papules, and vesicles (++) Erythema, papules, and numerous confluent vesicles/blisters (+++) [57]
Photopatch	Patch test in duplicate One area exposed to 5 joules/cm^2^ of UVA irradiation for 20–30 min Readings: at 48 h (before irradiation) and 72–96 h Result: comparison between both areas
ROAT	FDE or high clinical suspicion with negative results in the regular patch test Applied in post-lesioned skin sometimes following local tape stripping Same or higher allergen concentration than previous negative test Application every 24–48 h until a positive result is obtained

The technique of repeated open application test (ROAT) is a variation of the patch test used to diagnose FDE or in cases of high clinical suspicion with negative results in the regular patch test (Table 1) [122].

- **Value of epicutaneous tests according to the drug and clinical entity**

Based on the initial reaction, patch tests present different diagnostic values. For maculopapular exanthema (MPE), it is considered useful, with 10–40% positive results. For SDRIFE and AGEP, the positive results increase to 52–82% and 50–58%, respectively. For reactions induced by ultraviolet exposure, mainly by NSAIDs, Photopatch is considered a valuable diagnostic test [57,67]. In other entities, such as DRESS and SJS/NET, the value differs depending on the drug evaluated. In DRESS, there is a good sensitivity reported when testing carbamazepine (57–100%) and amoxicillin (44–100%); for allopurinol, however, the diagnostic value is very low [67,123]. In SJS/TEN, with a lower sensitivity in general, the most reliable drug to test is carbamazepine, with 64% positive results, followed by antibiotics, with 20% positive results reported [121,124].

- **Safety of epicutaneous tests**

Globally, patch testing is a safe technique. However, based on the drug (anti-tuberculosis drugs, beta-lactams, and corticosteroids, among others), the initial clinical entity, and the patient’s conditions (e.g., immunodeficiencies such as HIV), it has, in some cases, reproduced/reactivated the initial reaction. For severe reactions such as SJS/TEN, a graded concentration test is also recommended, starting at 1% concentration and increasing to 10% [116].

#### 4.3.3. Drug Challenge Tests

If in vitro tests and epicutaneous/skin tests are negative, a drug challenge with the suspect agent might be the only way to discard or confirm its imputability. Drug challenges are considered the gold standard for diagnosing or ruling out a drug allergy [101].

They are based on the patient’s exposure to the suspected drug in incremental doses, always in a medically supervised environment. Additionally, it can be helpful in de-labeling patients without a previous reaction or an unsuggestive history of drug allergy or when a multiple drug allergy is reported. When a culprit drug is identified, a drug challenge can be used to confirm tolerance to other drugs of the same group, ruling out cross-reactivity. A risk assessment must be performed first in order to evaluate the risk/benefit ratio, given that the drug challenge must be performed when the allergy probability is very low (non-suggestive anamnesis and/or negative result in validated allergy tests previously performed) [101]. This technique can be performed in one or two drug doses. In a one-step (dose) challenge, the total dose is administered in a single dose. In the two-step challenge, the total dose will be divided between a first dose of 10% to 25% of the target dose in a first step and the rest in a second step with a time interval between 30 min and 2 h, based on the initial reaction chronology. A placebo control challenge before this test can be performed when patients refer primarily subjective symptoms or for those who report multiple drug allergies [101]. Prior to a drug challenge, the patient must sign a consent document.

Although there are major contraindications for a drug challenge, such as previous SCARs or organ-specific reactions [101], there are exceptional circumstances in which challenges can be performed, always balancing risk and benefit [26,42,43,108].

Intravenous challenges before a drug desensitization to chemotherapy or biological treatments have been shown to be useful for reducing the number of procedures, preventing 24% of unnecessary desensitization [125]. To learn more about safety and efficacy of desensitization protocols, the readers can consult the following reference [101].

Performing the challenge under the direct supervision of an expert allergist in a specific and well-equipped facility after a conscious anamnesis and negative test results is crucial for maintaining the safety effectiveness of the procedure [101].

The use of drug challenges to de-label patients with allergy has become a crucial tool to prevent unnecessary alternative broad-spectrum antibiotic prescriptions, which favors the development of antibiotic resistance [126].

In children, a direct challenge without prior skin testing has been proposed to study mild exanthema associated with infectious diseases and beta-lactam treatments based on the frequency of infectious mild exanthema not caused by the antibiotic treatment [127,128]. Several cohorts have reported the safety of this diagnostic pathway in children, with a rate of negative challenges higher than 95% of cases, including the study of immediate and delayed reactions [129]. Compared with the safety rate when oral challenges with beta-lactams are performed after negative skin testing, it is similar (3.4% vs. 2.4%, respectively), endorsing this practice [130].

In the adult population who reported penicillin allergy, an oral challenge without prior skin testing has also been evaluated. The urgent need for a beta-lactam treatment in hospitalized patients a direct drug challenge can be justified in specific cases and under specialized supervision by well-trained allergy physicians. It is estimated that 75–90% of the penicillin allergy labels in hospitalized patients are not real allergies, and half can be ruled out with a direct oral challenge [131]. Today, in adult populations, drug challenges without previous skin testing are not recommended in low-risk adults with non-immediate reactions other than palmar exfoliative exanthema [61]. However, there is an effort toward better drug allergy management led by allergists, searching for strategies to improve the efficiency of diagnostic drug allergy tools. In line with this, a penicillin allergy clinician decision rule called PEN-FAST has recently been proposed to identify the candidates to best undergo a direct challenge, with an NPV of 96.3% [132].

Before a drug challenge, it is mandatory to perform careful patient selection and optimal risk assessment, with a clear action plan in case of reaction, and for it to be led by an expert allergist in the field to ensure patient safety. Even if more research is needed to standardize safety, the already published incipient data on direct drug challenges, including various drugs, are encouraging and always under the direct supervision of well-trained allergy physicians.

### 4.4. Value and Limitations of In Vitro Tests

In vitro tests have the advantage over in vivo diagnostic tests of being safe. Moreover, several drugs can be tested simultaneously. These tests can only be conducted by specialists in the field.

There are two categories of in vitro tests commonly used in the diagnosis of immediate DHRs: (1) histamine and tryptase determinations, which are not drug specific and are markers of type I hypersensitivity in general; and (2) identification of drug-specific IgE, and the basophil activation test, which are drug specific and can help to identify or confirm drug causality [133,134,135].

Regarding non-immediate reactions, in vitro tests are available mainly for drug causality evaluation in type IV reactions. Hence, in vitro tests are aimed at the identification of drug-specific T cells and are based on the property of antigen-specific T cells being activated upon stimulation with the nominal antigen in sensitized patients [136]. These tests measure well-known antigen-specific T cell responses (such as proliferation, cytokine production, and soluble mediators, or identification of cell membrane activation markers) in the patient’s peripheral blood lymphocytes after in vitro stimulation with the suspected culprit drug or its metabolites [133,137]. Proliferation and cytokine secretion-based assays will be discussed because these are the tests most frequently used.

#### 4.4.1. In Vitro Assay for Detection of Specific IgE

IgE antibodies with specificity toward certain drugs are the molecules responsible for immediate type I hypersensitivity reactions. These reactions occur within 1 to 6 h after contact with the drug. Specific IgE (sIgE) is produced against a hapten–carrier conjugate during an asymptomatic sensitization phase. Subsequent attachment to high-affinity IgE receptors on mast cells and basophils and renewed contact and cross-linking of sIgE lead to cell activation and release of several mediators (histamine, tryptase, leukotrienes, and others) that cause the symptoms.

- Procedure

The most available commercial method to measure drug–sIgE antibodies in serum is the fluoroimmunoassay, in which the drug is covalently bound to spacers on a solid phase with high surface capacity. Quantification of drug–sIgE is more complicated than that of sIgE to protein allergen extracts because drugs and their metabolites are low-molecular-weight haptens, which must be bound to a carrier to become immunogenic. Despite technical difficulties for suitable chemistry, antigenic determinants for binding to IgE could involve a drug–carrier structure not present in the assay. Quantification relies upon measurement of drug–hapten–carrier antibody complexes [134,135].

- Value and utility

Measurement of sIgE with determination of mediators during the acute phase of an immediate DHR has the potential to support DHR diagnosis, confirm a culprit drug, and avoid a drug provocation test. Advantages of the sIgE assay in comparison with other in vitro methods, such as cellular assays, are that serum samples can be frozen and stored for later use, they can be transported more easily to other laboratories, and the assays can be automated. Furthermore, they are risk-free for the patient [114].

According to published studies, the accuracy of the sIgE assay by ImmunoCAP depends on the drug involved; however, sensitivity tends to be variable (0–85%) for beta-lactam allergy [138] and is heterogeneous for neuromuscular blocking agents (NMBAs), ranging from 83–92% for rocuronium, 78–84% for morphine, and 44% for suxamethonium in NMBA allergy. Good sensitivity has only been described for chlorhexidine, alpha-gal, latex, and some non-commercially available NMBAs. The low sensitivity of sIgE to drugs could be a result of the drug binding to a solid phase, the dependence on a carrier as part of the antigenic determinant, an inadequate density of haptens in the conjugate, or that drug metabolites, which are not always included in the assays, are the main allergens [114].

Regarding specificity, it is generally good, although drug dependent. For example, sIgE measurements from morphine, a biomarker for sensitization to substituted ammonium structures, are not consistent with the clinical presentation, with most sensitized patients presenting with no clinical symptoms. In some cases, the reason for false positive results relates to high titers of total IgE, resulting in nonspecific binding to morphine [139]. High levels of total serum IgE can also induce false positive results to beta-lactams, and the application of drug sIgE/total IgE ratio can improve specificity [140].

In some patients with suspected IgE-mediated hypersensitivity to penicillin and a positive ImmunoCAP result, sIgE to penicillins can be directed to a cross-reactive epitope, phenylethylamine, an allergenic structure related to penicillin but different from the major and minor allergens.

- Limitations

Only a limited number of drug-specific sIgE immunoassays are available, and these assays are much less validated than those directed against allergenic proteins. Thermo Fisher, a commercial provider of sIgE assays (ImmunoCAP, Thermo Fisher, Uppsala, Sweden), markets sIgE tests for the drugs penicilloyl G, penicilloyl V, ampicilloyl and amoxicilloyl determinants, cefaclor, chlorhexidine, chymopapain, bovine gelatin, human insulin, morphine, pholcodine, and suxamethonium only. Moreover, ImmunoCAP Special Allergen Service tests are for research use only (2023): atracurium, cellulase, methylprednisolone, pancreatin, penicillin minor determinant, polyhexanide, propyphenazone, protamine, and rocuronium (Thermo Fisher ImmunoCAP Catalog 2022). Furthermore, the correlation between sIgE and skin test results from drugs has been found to be generally poor [141]. In addition, sensitivity of sIgE assays decreases over time. It has generally been recommended to perform a specific allergy workup, if possible, 4–6 weeks after complete resolution of all clinical symptoms and signs, given that after a time interval of more than 6–12 months, some drug tests have already turned negative [2].

#### 4.4.2. Serum Tryptase Measurement

Tryptases are mast cell-specific serine proteases and are the most abundant of the mast cell proteases. The immature isoforms (pro-α tryptases and pro β-tryptases) are monomers constitutively exocytosed, which account for baseline serum tryptase (BST) levels. The mature forms are homotetramers (β-tryptase) and heterotetramers (αβ: αβ-tryptase), which are stored in secretory mast cell granules in association with proteoglycan scaffolds containing heparin that are released as a consequence of mast cell activation during the process of degranulation [142]. Tryptase has a short half-life (90–120 min); thus, it should be measured between 30 min and 2 h after the onset of symptoms. To determine BST for comparison, either a recent value or a sample obtained at least 24 h after the resolution of symptoms is taken. The tryptase is considered elevated if serum concentrations are ≥1.2 × [basal tryptase value] + 2 μg/L. This formula detects drug hypersensitivity reactions with the best sensitivity and specificity [134]. Recently, a new algorithm has been proposed to take into account the variability in BST values. It is available as an online calculator at https://triptase-calculator.niaid.nih.gov (accessed on 7 August 2023) [143].

- Principle of the procedure

The ImmunoCAP fluoroenzymatic assay for total tryptase (mature plus immature forms) is currently the only commercial in vitro diagnostic test for clinical practice (Phadia Thermo Fisher, Uppsala, Sweden). The assay is robust, and the tryptase in serum is stable at room temperature [134]. It can be performed on multiple sample tubes (e.g., without anticoagulant or with heparin or ethylenediaminetetraacetic acid), transported at room temperature, and stored refrigerated for up to 1 week without affecting the performance of the in vitro diagnostic test. Anti-tryptase, covalently coupled to ImmunoCAP, reacts with the tryptase in the patient’s sample. After washing, enzyme-labeled antibodies against tryptase are added to form a complex. Unbound enzyme–anti-tryptase is washed away, and the bound complex is then incubated with a developing agent. After stopping the reaction, the fluorescence of the eluate is measured. The higher the response value, the more tryptase present in the sample. The responses for the patient samples are transformed to concentrations with the use of a calibration. The total tryptase assay is not subject to interference from hemolyzed, lipemic, or icteric blood samples. (ImmunoCAP tryptase fluoroenzyme immunoassay; Directions for Use 52-5467-EN/02).

- Value and utility

Tryptase is used as a biomarker for the diagnosis, stratification, prognostic evaluation, follow-up, and therapeutic evaluation of multiple mast cell-related conditions, including diagnostic confirmation of severe immediate hypersensitivity reactions (anaphylaxis) and prognostic evaluation of the risk and severity of immediate hypersensitivity reactions, irrespective of the trigger [142]. Tryptase levels reflect total mast cell numbers in the body plus mast cell activation levels. It can be determined at the acute phase of reaction and allows the assessment of mast cell involvement. Tryptase measurements are critical to the diagnosis of perioperative anaphylaxis because clinical criteria might not be applicable in that setting. To do so, two serum tryptase samples with adequate timing are mandatory. Adequate timing is 30 min to 2 h after the onset of symptoms. Blood collection 24 h after the resolution of all symptoms is necessary for basal measurements [144].

- Limitations

Tryptase levels after a drug hypersensitivity reaction should be compared with basal values. The basal values are increased by obesity, older age, chronic helminthiasis, and decreased kidney function and are strongly elevated in mastocytosis. Baseline tryptase above 8.4 μg/L must raise the clinician’s awareness of the potential contribution of hereditary α-tryptasemia and other mast cell-related disorders to the clinical presentation and risk assessment of patients experiencing immediate hypersensitivity reactions [142]. Elevated tryptase concentrations are not a primary marker of an immediate drug hypersensitivity reaction but of a mast cell activation and degranulation, which can, in part, support the diagnosis. Serum tryptase levels are increased mainly in more severe immediate-type reactions, such as anaphylaxis, whereas it is less helpful in differentiating mild allergic or non-allergic reactions [134].

#### 4.4.3. In Vitro Histamine Release

Histamine is produced by decarboxylation of histidine present in the Golgi apparatus of mast cells and basophils, and large amounts of this compound are stored in cell granules. Histamine is released extracellularly via degranulation upon basophil and mast cell activation. It is a key mediator of type I hypersensitivity [145]. Histamine is secreted within minutes of the anaphylactic reaction but has a very short half-life in blood, given that it is metabolized only 30–60 min after release by histamine transferase [146].

- Procedure

Histamine levels can be measured with commercial immunoassays. Because of its short half-life, samples should be collected within 15–20 min of the reaction onset, kept refrigerated, processed as quickly as possible, and should not be hemolyzed [145]. An indirect method for the histamine determination consists of measuring its metabolites (N-methylhistamine or N-methylimidazoleacetic acid) in urine. These appear within 30–60 min of the event and remain detectable for a 24-h period after the reaction starts [18]. Histamine release can also be quantified by flow cytometry, although this technique is not clinically validated. It is designated Hista Flow, and the intracellular content of histamine and its release are analyzed by an enzyme affinity method using the histaminase diamine oxidase [147].

- Value and utility

Histamine levels are elevated at the acute phase of reaction, and they can be determined by the assessment of mast cell and/or basophil involvement. A sensitivity ranging from 61–92% and a specificity ranging from 51–91% have been reported for plasma histamine tests in anaphylaxis diagnosis [114]. Circulating histamine levels tend to correlate with the severity of the anaphylactic reaction and are more likely to be increased than tryptase levels, especially in less severe cases [148].

- Limitations

The short half-life of histamine prevents its use as a reliable marker of anaphylaxis. Furthermore, due to significant inter- and intraindividual variability, levels measured during a reaction should also be compared with baseline levels for accurate interpretation (pre- and/or post-reaction). Other disadvantages include the following: (a) given that histamine is also produced by neurons and bacteria, increased histamine does not necessarily indicate mast cell/basophil activation; (b) histamine levels can be influenced by food intake, drug intake, or both; and (c) measurement methods have specific requirements and are expensive. However, histamine assay at 30 min after a suspected hypersensitivity reaction is recommended by French guidelines. This recommendation appears to be based on evidence that the diagnostic accuracy of perioperative hypersensitivity (POH) is increased when histamine and tryptase assays are combined. Although the significance of histamine assay in the diagnosis of POH is controversial, there is no reason not to measure histamine levels if facilities for the measurement are available [149].

#### 4.4.4. Basophil Activation Test

The basophil activation test (BAT) is a flow cytometry assay that detects the ability of IgE to activate basophils, which are stimulated due to drug exposure. The BAT measures the expression of activation markers (mainly CD63 and/or CD203c) on the basophil membrane following cross-linking of IgE antibodies caused by a drug. BAT avoids exposure of patients to the drug being investigated, thus making the diagnostic process safer for the patient. CD63 is normally expressed on the inner side of the granule membrane, and it can be detectable after fusion of the intracellular granules with the cytoplasmic cell membrane during basophil degranulation and mediator release. CD203c has low expression on the cytoplasmic cell membrane in resting basophils and is upregulated after cell activation [150].

- Procedure

BAT is typically performed using whole blood anticoagulated with heparin. Given that a decrease in reactivity is observed over time, blood must be used within 24 h of collection. An aliquot of blood is incubated at 37 °C with the suspicious drug, using previously standardized concentrations and avoiding cytotoxic effects. Basophils are identified in the flow cytometer as side scatter low, CD123c+, CD193+, HLA-DR- cells. The gates of the activation markers CD63 and/or CD203c are set on the negative control, and basophil activation is measured above these gates for the experimental conditions with various concentration of the suspected drug or positive control. It is also important to assess whether the basophils respond to an IgE stimulus, such as anti-IgE or anti-FcεRI. Approximately 10% of the population are basophil non-responders to IgE. This is important because in non-responding individuals, BAT should be considered not interpretable. These patients usually have positive skin test responses, indicating mast cell but not basophil responsiveness [151].

The standard positive threshold that is empirically adopted for positive results is more than 5% CD63+ basophils [152,153]. The BAT can also be considered positive if a two-fold increase in the stimulation index (SI) is observed. The SI is calculated by dividing the geometric mean fluorescence intensity (gMFI) of CD63 and/or CD203c expression on basophils after stimulation with the drug by the gMFI of these markers on basophils stimulated with saline [154].

- Value and utility

The BAT can be extremely useful in the case of life-threatening drug allergies in which patients cannot be re-challenged, in case of drugs for which no other tests are available, or their results are equivocal before considering provocation tests [155]. BAT should be considered in drug allergy diagnosis if the drug is known to produce false positive results in skin testing; if there is no drug source to use for skin or sIgE testing; if there is discordance between the patient history and sIgE or skin tests; if the symptoms in the patient history suggest that skin testing could result in systemic response; and before considering a provocation to confirm the causative drug [155].

The application of BAT in drug allergy goes further than standard diagnosis to possibly serving as a biomarker for anaphylaxis following drug desensitization. Drug desensitization is imperative for patients with allergy requiring full therapeutic doses of lifesaving medication. For instance, BAT has been used to successfully identify patients allergic to platinum compounds who have high risk of adverse reactions during drug desensitization, with increased CD203c expression being indicative [156]. Furthermore, BAT can be used to test potentially non-cross-reactive alternatives, preventing new reactions [157].

The sensitivity of BAT for drug allergies is lower than that for food allergies; for example, 55% for beta-lactams [158] and 80% for rocuronium [159]. Sensitivity is also influenced by the activation marker measured (CD63 or CD203c). In patients with quinolone-induced immediate DHRs, the best sensitivity–specificity was obtained by using CD203c for moxifloxacin and CD63 for ciprofloxacin [160]. The use of both activation markers is recommended to improve sensitivity.

BAT is only useful for allergic drug hypersensitivity reactions (IgE-mediated pathogenesis). It is not useful for evaluation of non-allergic hypersensitivity to NSAIDs.

- Limitations

Individuals being tested should stop treatment with oral steroids or immunosuppressant drugs (ciclosporin A) 3 weeks before the test. A negative result can be obtained if it is performed within 1–2 weeks after a recent reaction (refractory period) or if the last reaction took place more than 1 year previously. On the other hand, false positive results can be due to high/irritating doses of the drug, leading to nonspecific basophil degranulation [157]. Antihistamines and topical treatments with steroids do not influence the BAT result [161]. The use of BAT in clinical practice requires analytical validation of the methodology, clinical validation of the test against the patient’s phenotype, and continued quality assurance [153].

#### 4.4.5. Lymphocyte Transformation Test

- Definition

The lymphocyte transformation test (LTT) relies on the ability of drug-specific memory T cells to proliferate upon stimulation with the nominal antigen. This proliferation assay was initially named LTT because antigen-stimulated T cells undergo blastogenesis upon cell stimulation, and the old technique readouts for the assay relied on cell observation under the microscope. This test is indicated mainly in non-immediate allergic reactions, specifically in type IV T cell-mediated reactions. LTT is recommended at least 4 weeks after completion of corticoid treatment [162].

- Procedure

Detailed technical aspects of the LTT have been described [146,163]. In short, peripheral blood mononuclear cells (PBMCs) from the patient are stimulated with the suspected drugs for 6 days, and the proliferation is evaluated through the incorporation of ^3^H-thymidine to newly synthesized DNA. An SI is calculated as the ratio of ^3^H incorporated by drug-stimulated cultures and basal ^3^H incorporation by unstimulated cells. An SI ≥ 2 is usually considered positive, with some exceptions (Table 2). The fluorescent dye 5,6-carboxylfluorescein diacetate succinimidyl ester (CFSE) dilution with flow cytometry analysis is used as an alternative to classical LTT to assess drug-stimulated T cell proliferation [163]. It allows the discrimination of proliferative responses in specific subpopulations, although it has not been widely applied for diagnostic purposes.

- Value and Utility

The reliability of a test depends on its sensitivity and specificity. A number of studies have aimed to establish the utility of LTT in the diagnosis of drug allergy [165,166,167]; however, there is controversy over the specificity and sensitivity of LTT in general. Different “gold standards” have been used to calculate the sensitivity and specificity of LTT. The clinical history, skin tests, or controlled administration of the drug have been used as the reference methods to estimate the likelihood of true drug sensitization [165]. Although a drug provocation test is considered the best gold standard for the identification of the drug eliciting a hypersensitivity reaction [2], there are many limitations of a provocation test that must be considered, and the test can give false positive and false negative results [168]. Additional difficulties for sensitivity and specificity assessment arise from the lack of standardization regarding drug concentrations tested by each laboratory. Accordingly, significant variability has been reported in various series, which could be due to the previously mentioned issues as well as the heterogeneity in the clinical entities and drugs tested [28,165,166,167,169,170].

Overall, good sensitivity (58–89%) and specificity (93–100%) have been reported for mild and moderate drug hypersensitivity reactions.

LTT has been shown to be frequently positive in maculopapular exanthema, DRESS, and AGEP cases [171], with LTT sensitivity higher compared with cutaneous tests [136,165].

In DRESS, most information is derived from small studies involving few patients or case reports [109,172,173,174,175,176,177,178,179,180,181,182]. Pichler and Tilch reported positive LTT results in more than 50% of DRESS cases [162]. A review analysis of cases published up to 2017 calculated an overall sensitivity of 67% for DRESS proliferation assays, with 90% specificity [183]. In a more recent study involving 41 DRESS cases employing various medications, the reported sensitivity and specificity when the LTT was performed in resolution samples were 72% and 83%, respectively, when investigating related and non-related drugs evaluated according to the Spanish Pharmacovigilance System causality algorithm [52]. In this study, higher sensitivity (S) and specificity (Sp) values were found for determined groups such as anticonvulsants (S 100%, Sp 100%), anti-TB drugs (S 87.5%, Sp 100%), and beta-lactam antibiotics (S 73%, Sp 100%). A recent study determined 91% sensitivity, 91.67% specificity, 91% PPV, and 91.67% NPV for the LTT with vancomycin performed in 14 vancomycin-induced DRESS cases and 12 vancomycin-tolerant control donors [164].

Although the LTT has been used for more than three decades to investigate drug sensitization in non-immediate reactions, its usefulness in SJS/TEN is still controversial. Although the LTT is traditionally performed upon resolution of the clinical symptoms because it identifies drug-specific memory T cells that develop after the acute immune response, some authors have reported low LTT sensitivity in SJS/TEN [184,185] and have suggested that LTT sensitivity might increase if the test is performed in the acute stage of SJS/TEN. However, only small case series were included in these studies, with low representation of highly suspected drugs with previously well-established assay conditions and low threshold considered for positivity (SI > 1.8) [176].

In a recently published article involving 26 well-defined SJS/TEN cases studied after recovery, positive LTT results were obtained in 80% of SJS/TEN cases with 86.4% sensitivity, 73.5% specificity, 67.9% PPV, and 89.3% NPV using ALDEN as the gold standard for causality assessment of 56 drugs taken by patients [55]. These results suggest that the LTT is an effective approach after recovery and could be included among the workup tools for ascertaining drug causality in patients with a past history of SJS/TEN. In the same study, eighteen drugs were investigated in seven SJS/TEN patients during the acute reaction, with only 33% sensitivity and 58.3% specificity.

Regarding LTT utility in AGEP, available information is derived from small studies [172,186,187,188,189] or case reports [190,191,192,193,194,195,196,197,198,199]. In the largest series published involving thirteen patients, only seven (54%) cases were positive [200]. The absence of appropriate controls and the scarce publication of negative data preclude the estimation of sensitivity and specificity of LTT, specifically in AGEP [183].

- Limitations

The LTT requires a specialized laboratory and skilled personnel. A consensus exists among most laboratories regarding protocol and cutoff for positivity [162]. Multiple publications addressing specific T cell responses to frequently involved drugs provide useful drug concentrations for in vitro testing. Standardization regarding optimal drug concentrations is lacking for many compounds, particularly for newly marketed drugs. In addition, the long incubation period (6 days) precludes the evaluation of cytotoxic compounds such as those used for chemotherapy in oncologic patients. Immunosuppressive drugs such as corticosteroids are also inhibitors of T cell proliferation and limit the usefulness of LTT in those patients with chronic steroid treatment.

When CFSE dilution is used as readout for cell division, larger amounts of blood samples are needed if several concentrations of different drugs need to be tested. On the other hand, the low frequencies of responding cells require experienced personnel for the analysis of flow cytometry results to accurately discriminate the specific signal from background noise. Few reports have been published, and no consensus exists regarding the analysis and calculation of stimulation indices for CSFE dilution in various lymphocyte subpopulations.

#### 4.4.6. Enzyme-Linked Immunosorbent Spot Assay

- Definition

Proliferation tests have been recently complemented by cytokine-production assays, among which the technique known as an enzyme-linked immune absorbent spot assay (ELISpot) is considered one of the most sensitive to detect antigen-specific T cells through the detection of soluble factors released upon T cell stimulation. This technique quantifies the activation of drug-specific cells by determining the number of spot-forming units (SFUs), equivalent to spot-forming cells, that release cytokines or cytolytic molecules after the patient’s PBMCs are activated in vitro with the suspected drug(s). The ELISpot has recently been used as an alternative to LTT, mainly through the assessment of IFN-γ–releasing T cells (IFN-γ–ELISpot) [201,202,203].

- Procedure

The patient’s PBMCs are added to a 96-well plate coated with specific anti-cytokine capture antibody. In drug-induced delayed hypersensitivity, IFN-γ is mostly used, although other cytokines or cytotoxic proteins can be evaluated. A polyclonal T cell stimulus is used as a positive control. The background immunological activation can be assessed with negative controls (cells and media). Cytokine secretion is captured by the anti-cytokine antibodies in the next 24–48 h. Detection antibody and enzyme substrate are added just before reading the plate. The SFU, representing cells that secrete cytokines, are then identified and counted [204]. Diverse cutoff points are considered for positivity in various reports, some considering a positive response as greater than or equal to 50 SFU/million PBMCs after background (unstimulated control) removal [189], whereas lower thresholds for positivity were considered in other studies [201,205,206].

- Value, Utility

The sensitivity of ELISpot was 91% in a group of 22 well-selected patients with MPE due to amoxicillin, with 100% specificity in 20 controls tested [201]. Besides the elimination of radioactive reagents, an additional advantage of ELISpot in relationship with LTT is shorter time for the stimulation of drug-specific T cells (6 days for LTT versus 24–48 h for ELISpot). An additional advantage of cytokine release assays is that they are less dependent on corticoid treatment, which increases the rate of positive results during the acute phase in patients under steroid treatment. Accordingly, recent reports have shown good sensitivity and specificity of ELISpot–IFN-γ when it is performed in acute samples from patients with SCARs [206,207]. In this regard, some authors have suggested that the optimal approach for investigation of putative culprit drugs would be ELISpot–IFN-γ analysis in acute samples and LTT after resolution of the adverse reaction [205].

In SJS/TEN cases, the sensitivity was 50% in various reports, including a limited number of cases [205,208]. A systematic review of 28 tests performed in SJS/TEN cases found 71% sensitivity and 96% for IFN-γ–ELISpot to various drugs [183].

Although different cytokines might predominate in different clinical entities, and Th2 cytokines appear to predominate in DRESS, positive IFN-γ–ELISpot assays have been reported in DRESS cases [209,210]. More interestingly, nine out of thirteen allopurinol-induced DRESS cases have been reported with positive IFN-γ–ELISPOT to the primary metabolite oxypurinol, whereas 20 of 21 control donors tested negative [206]. However, although cytokine-releasing tests appear to be useful in the diagnosis of bullous reactions and in increasing sensitivity and maintaining high specificity, a systematic review of ELISpot measuring IFN-γ did not appear to significantly improve sensitivity in patients with DRESS compared with LTT alone [183]. On the other hand, analysis of the results in nine DRESS cases studied in the acute phase estimated a sensitivity of 77.8% [205]. More recently, in a study involving 34 cases tested at various time points, the sensitivity of the IFN-γ release ELISpot assay for DRESS was estimated at 56%. The same study estimated the sensitivity of the assay for severe MPE at 53% (N = 17) and the specificity at 100%, considering the absence of positive results in five control donors tested. Of note, a higher threshold for positivity (50 SFU/million cells) was considered in this study [208]. Additional studies are needed to clarify its value in acute and resolution samples from DRESS cases.

Regarding AGEP cases, IFN-γ production upon drug stimulation has been reported in small AGEP series [202,205,207] with different results. Whereas results were positive in 100% of four AGEP cases analyzed in the acute phase [205], only 40% of the cases tested positive in five patients analyzed at various time points [208].

Given that a distinct cytokine microenvironment has been described for AGEP, other cytokines have been tested in ELISpot assays. IL-36α release has also been detected [200]. A recent publication analyzed the production of IL-22 in acute samples from nine cases upon exposure to the culprit drug and found eight positive patients [211].

- Limitations

For the time being, there are no accepted standard criteria, or even a common attitude, as to how to discriminate between positive and negative ELISpot assay responses in suspected DHR. In addition, some parameters that determine a positive spot, such as the threshold for size or intensity, are arbitrary or depend on automated visual analysis equipment [212]. Therefore, the quantification of results can be somewhat subjective and could affect inter-laboratory reproducibility.

Differences in laboratory protocols, similar to the issues found for LTT assays, and in the criteria used in the assessment of ELISpot plates, along with the issue of technical feasibility and reproducibility, could limit the use of this assay in the routine diagnosis of drug hypersensitivity reactions.

#### 4.4.7. Cyto-Lymphocyte Transformation Test

- Definition

A new test has recently been developed, the Cyto-LTT, using a bead flow cytometry immunoassay and presumably easily feasible for routine diagnosis. This test combines the measurement of cytokines (IL-5, IL-13, and IFN-γ) and cytotoxic markers (granzyme B and granulysin) [137].

- Procedure

Just as LTT assays, PBMCs from the patient are stimulated with the suspected drugs for 6 days in round-bottom 96-well plates. The culture supernatants are collected thereafter, and cytokine/soluble mediator content is evaluated in a multiplex bead assay by flow cytometry [185].

- Value and utility

Few cases have been published. It has shown higher sensitivity (>80%) than LTT while maintaining good specificity in patients with MPE, AGEP, and DRESS, with similar results among various tested drugs [168,178]. Among the various cytokines tested, IL-5 results appear to be more valuable in assessing culprit drugs in DRESS cases, consistent with its relevance in the physiopathology of this condition [213]. These tests might also be helpful for identifying the culprit drug in SJS/TEN; however, consistent data are still lacking, and further studies are needed to confirm those promising results [168].

- Limitations

Similar to conventional LTT, this assay requires a specialized laboratory and skilled personnel for careful sterile cell culture. No consensus exists regarding cutoff cytokine levels for positivity. Standardization regarding optimal drug concentrations is lacking for many drugs. The long incubation period (6 days) precludes the evaluation of chemotherapeutic medications in oncologic patients. However, the test is less dependent on corticosteroids than the proliferation assay in a conventional LTT.

#### 4.4.8. Human Leukocyte Antigen Determinations

- Definition

An increasing number of studies have shown an association between specific HLA alleles and the development of delayed drug hypersensitivity reactions. This topic has been exhaustively reviewed [30,38,214]. Particularly, strong HLA class I associations have been found for drug hypersensitivity to abacavir, carbamazepine, allopurinol, dapsone, and vancomycin. Among them, HLA-B*57:01 has been found to be associated with abacavir hypersensitivity in most ethnic populations [215,216]. For carbamazepine-induced delayed hypersensitivity reactions, the most powerful association has been established with HLA-B*15:02 as a genetic risk factor in developing SJS/TEN in Han Chinese [217], Thai, Indian, and Malaysian populations [218]. On the other hand, HLA-A*31:01 has also been associated with carbamazepine-induced MPE/DRESS in Japanese, Han Chinese, and Europeans [219,220,221]. HLA-B*58:01 allele is associated with relatively high risk of allopurinol-induced MPE, DRESS and SJS/TEN in Han Chinese and other ethnic populations such as Thai, Japanese, Koreans, and Europeans [30,222,223]. Dapsone-induced hypersensitivity syndrome has been found to be strongly associated with HLA-B*13:01 in Han Chinese, Thai, Taiwanese, and Malaysian patients [221,224], and HLA-A*32:01 has been identified for vancomycin in European patients [203].

Well-defined HLA–drug–phenotype associations in various populations are summarized in Table 3.

- Procedure

Genomic DNA from patients with drug reactions can be extracted from whole blood or saliva. HLA genotyping is classically based on reverse sequence-specific oligonucleotide–polymerase chain reaction using genomic DNA. However, various techniques can be used, such as high-resolution HLA class I and II typing with next-generation sequencing methods. To facilitate HLA testing with rapid turnaround times, cost-effective single allele assays have been developed for many class I HLA alleles, such as HLAB*58:01, HLA-B*15:02, and HLA-A*32:01 [225,226,227].

- Value and utility

HLA testing prior to drug administration can be used as a tool to prevent severe hypersensitivity reactions in patients at risk. The number of individuals needed to test for a specific HLA allele to prevent one case of drug hypersensitivity is dependent not only on the PPV of the HLA risk allele but also on the carriage rate of the risk allele and the prevalence of the phenotype in the population. The PPV varies among hypersensitivity phenotypes and can be as high as 55–58% for HLA-B*57:01 in abacavir hypersensitivity, with 100% NPV. For most phenotypes, however, the PPV is quite low (2% for HLA-B*58:01 in allopurinol SCAR or for HLA-B*15:02 in SJS/TEN to CBZin Han Chinese) [210]. It has been calculated as 20% for HLA-A*32:01 in North American patients of European descent with vancomycin-induced DRESS [203]. However, considering the prevalence of the disease among European patients and the average carriage rate of HLA-A*32:01 at 5%, a lower PPV is likely more realistic [221] and consistent with the results recently reported in Spanish patients [164].

The usefulness of HLA testing has been documented in various populations for a selected set of drugs. Among them, the paradigm is HLA testing in patients with HIV prior to treatment with abacavir. With 100% NPV, 55% PPV, and a prevalence of HLA-B*57:01 in 4% to 10% of individuals in occidental countries, only thirteen patients would need to be tested to prevent one case of clinically diagnosed abacavir hypersensitivity [228]. Accordingly, HLA-B*57:01 screening is recommended for abacavir by the European and American regulatory agencies (European Medicines Agency and Food and Drug Administration [FDA], respectively) before treatment, and a drop in the prevalence of abacavir hypersensitivity from 12–7.5% to 3–0% in several countries [229,230] has been demonstrated.

A prospective study in Taiwan including 4120 non-carriers of HLA-B*15-02 treated with carbamazepine and 215 patients carrying the risk allele with an alternative treatment confirmed the absence of SJS/TEN cases compared with the 10 expected cases [231]. Currently, genetic testing is performed in Asian countries such as Taiwan, Singapore, and Thailand [223]. The incidence of carbamazepine-induced SJS/TEN has decreased 92% in Singapore and 87% in Taiwan. A prospective screening of HLA-A*31:01 in Japanese patients before administration of carbamazepine showed a reduced incidence of SCARs [232]. In relationship with alopurinol-induced SCARs, prospective studies in Taiwan and Korea confirmed the utility of HLA-B*58:01 screening [233,234]. Also, in Asia, a clinical trial of prospective HLA-B*13:01 screening before prescribing dapsone proved significant prevention of hypersensitivity reactions in China [235].

Prospective HLA-B*15:20 and HLA*B58:01 screenings are covered by the national health systems in several Asian nations. The FDA recommends screening for HLA-B*15:02 before starting treatment with carbamazepine for at-risk patients, and screening for the presence of the HLA-B*58:01 allele is recommended by the American College of Rheumatology in individuals considered to be at high risk of developing allopurinol DHR. The implementation of guidelines and screening programs is nonetheless diverse in different countries according mostly to the prevalence of risk alleles and the patients needed to test to prevent one case [236,237].

- Limitations

Prerequisites for implementation of HLA testing prior to drug administration are that the associated HLA allele must be relatively frequent among the population and have a high NPV, the number of patients to test must be low, and the drug must have good efficacy, tolerability, and cost-effectiveness. HLA alleles have varied prevalence in different ethnic groups. Traditional HLA genotyping as a screening tool has not proven to be cost-effective for most drugs. However, allele-specific assays could be an alternative to consider when available.

## 5. Algorithms for Diagnosis of Immediate and Non-Immediate DHRS

**Figure 2:**
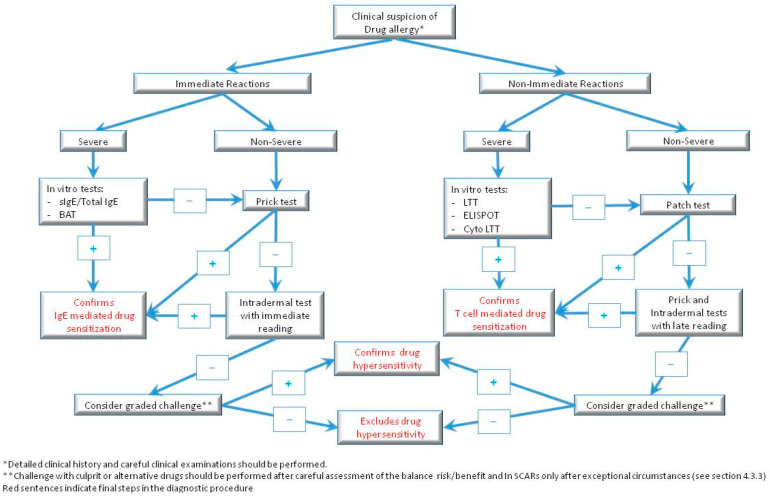
Algorithms for diagnosis of immediate and non-immediate DHRs.

The following algorithm summarizes the process for identifying the culprit drug after careful clinical evaluation of the drugs potentially involved. It merges the available in vivo and in vitro tests (Figure 2).

## 6. Conclusions and Unmet Needs

There is currently no diagnostic tool that offers 100% NPV for immediate or delayed hypersensitivity reactions, and any decision to reintroduce a drug or another member of its drug class in the treatment setting should weigh the risk/benefit ratio.

In vitro tests, such as sIgE determination and the basophil activation test, could complement the diagnosis of immediate reactions, particularly in severe reactions or in those for whom skin tests and drug provocation tests are risky. LTT and ELISpot are safe and valuable diagnostic techniques in SCARs, although they are only available in a few specialized centers.

Skin and patch testing show variable results depending on the clinical entity, the drug evaluated, and the time elapsed since the reaction, limiting the diagnostic utility of these techniques.

Regarding the drug provocation test, it has been shown to be effective to de-label and prevent unnecessary drug restrictions for patients. It should be performed when in vitro and skin tests have proven negative, only if the suspected drug is required to maintain the patient’s quality of life or for his/her survival, and after a SCAR has been discarded. However, current knowledge allows us to perform direct drug challenges in specific and selected cases. A well-trained drug allergy team led by an expert allergist and a well-equipped facility are crucial for maintaining the safety and effectiveness of the procedure.

A limitation of many studies is the small sample size. It is time for collaborative and multicentric studies that consolidate individual experiences with uncommon drugs or with rare and severe allergic drug reactions. These studies, with the inclusion of sufficient number of patients and exposed tolerant controls, would facilitate the harmonization of techniques, contributing to standardization of procedures and the ability to draw valuable conclusions.

## Figures and Tables

**Table 2:** Cutoff values for LTT positivity.

Drug	Result	SI ^1^
Beta-lactam antibiotics [162]	Negative	<3
	Positive	>3
Iodinated contrast media [162]	Negative	<4
	Positive	>4
Vancomycin [164]	Negative	<3
	Positive	>3
Other drugs [162]	Negative	<2
	Doubtful	2–3
	Positive	>3

^1^ SI: stimulation index.

**Table 3:** HLA associations in drug-induced hypersensitivity reactions described in different ethnic groups.

Drug	Clinical Entity	Risk HLA Allele	OR	PPV	NPV	NNT ^1^	Ethnic Risk Groups
**Abacavir**	Hypersensitivity	B*57:01	960	55%	100%	14	European, African American
**Carbamazepine**	SJS/TEN	B*15:02	>1000	3%	100% (Han Chinese)	1000	Han Chinese and Southeast Asian countries
**Carbamazepine**	DRESS/MPE	A*31:01	57.6	0.89%	99.98%	2857	European
		A*31:01	23.0	0.59%	99.97%	4000	Han Chinese
**Alopurinol**	SSJ/NET, DRESS, MPE	B*58:01	580	2%	100% (Han Chinese)	500	Han Chinese and Southeast Asian countries
**Oxcarbazepine**	SSJ/NET	B*15:02		0.73%	99.97%	1715	Han Chinese
**Dapsone**	DRESS	B*13:01	20	7.8%	99.80%	84	Han Chinese
**Vancomycin**	DRESS	A*32:01	70	20%		75	European descent

^1^ NNT Number of individuals needed to test to prevent one case.

## Data Availability

No new data were created.
